# The Diabetes Transition of Hospital Care (DiaTOHC) Pilot Study: A Randomized Controlled Trial of an Intervention Designed to Reduce Readmission Risk of Adults with Diabetes

**DOI:** 10.3390/jcm11061471

**Published:** 2022-03-08

**Authors:** Daniel J. Rubin, Preethi Gogineni, Andrew Deak, Cherie Vaz, Samantha Watts, Dominic Recco, Felicia Dillard, Jingwei Wu, Abhijana Karunakaran, Neil Kondamuri, Huaqing Zhao, Mary D. Naylor, Sherita H. Golden, Shaneisha Allen

**Affiliations:** 1 Section of Endocrinology, Diabetes, and Metabolism, Lewis Katz School of Medicine, Temple University, Philadelphia, PA 19140, USA; gpreethi7@gmail.com (P.G.); cherielisa.vaz@tuhs.temple.edu (C.V.); abhijanakaru@gmail.com (A.K.); nkondamuri@gmail.com (N.K.); shaneisha.allen@tuhs.temple.edu (S.A.); 2 Lewis Katz School of Medicine, Temple University, Philadelphia, PA 19140, USA; andrew.deak@temple.edu (A.D.); samanthawatts0711@gmail.com (S.W.); dprecco@bidmc.harvard.edu (D.R.); felicia.dillard@temple.edu (F.D.); 3 Department of Epidemiology and Biostatistics, College of Public Health, Temple University, Philadelphia, PA 19140, USA; tug30693@temple.edu; 4 Department of Biomedical Education and Data Science, Lewis Katz School of Medicine, Temple University, Philadelphia, PA 19140, USA; zhao@temple.edu; 5 School of Nursing, University of Pennsylvania, Philadelphia, PA 19104, USA; naylor@nursing.upenn.edu; 6 Division of Endocrinology, Diabetes, and Metabolism, Department of Medicine, Welch Center for Prevention, Epidemiology, and Clinical Research, Johns Hopkins University School of Medicine, Baltimore, MD 21205, USA; sahill@jhmi.edu

**Keywords:** rehospitalization, transition care, pilot study, prospective randomized trial

## Abstract

Hospital readmission within 30 days of discharge (30-day readmission) is a high-priority quality measure and cost target. The purpose of this study was to explore the feasibility and efficacy of the Diabetes Transition of Hospital Care (DiaTOHC) Program on readmission risk in high-risk adults with diabetes. This was a non-blinded pilot randomized controlled trial (RCT) that compared usual care (UC) to DiaTOHC at a safety-net hospital. The primary outcome was all-cause 30-day readmission. Between 16 October 2017 and 30 May 2019, 93 patients were randomized. In the intention-to-treat (ITT) population, 14 (31.1%) of 45 DiaTOHC subjects and 15 (32.6%) of 46 UC subjects had a 30-day readmission, while 35.6% DiaTOHC and 39.1% UC subjects had a 30-day readmission or ED visit. The Intervention–UC cost ratio was 0.33 (0.13–0.79) 95%CI. At least 93% of subjects were satisfied with key intervention components. Among the 69 subjects with baseline HbA1c >7.0% (53 mmol/mol), 30-day readmission rates were 23.5% (DiaTOHC) and 31.4% (UC) and composite 30-day readmission/ED visit rates were 26.5% (DiaTOHC) and 40.0% (UC). In this subgroup, the Intervention–UC cost ratio was 0.21 (0.08–0.58) 95%CI. The DiaTOHC Program may be feasible and may decrease combined 30-day readmission/ED visit risk as well as healthcare costs among patients with HbA1c levels >7.0% (53 mmol/mol).

## 1. Introduction

Hospital readmission within 30 days of discharge (30-day readmission) is a high-priority quality measure and cost target [1]. People living with diabetes are at higher 30-day readmission risk than those without diabetes [2,3,4]. Several interventions have shown promise for reducing the readmission risk of diabetes patients in mostly observational studies [4]. Selecting patients at high readmission risk for intervention may enable a more efficient use of resources than applying interventions broadly without regard to readmission risk. No previously published randomized controlled trials (RCTs) have tested an intervention designed to reduce readmission risk in patients with diabetes and high readmission risk as predicted by a validated tool. We previously reported on the development and validation of the Diabetes Early Readmission Risk Indicator (DERRI^TM^) [5,6,7], which predicts 30-day readmission risk in diabetes patients. The aim of the current pilot RCT was to explore the feasibility and potential efficacy of a novel, multi-component intervention, the Diabetes Transition of Hospital Care (DiaTOHC) Program, on 30-day readmission risk in adult patients with diabetes at high risk based on the DERRI^TM^.

## 2. Materials and Methods

### 2.1. Study Design, Setting, and Ethics

This was a non-blinded pilot RCT with two parallel arms that compared usual care (UC) to the DiaTOHC Program (Intervention) at Temple University Hospital, an urban, academic, safety-net hospital in Philadelphia, PA. The protocol was registered in the National Clinical Trials Registry (NCT03243383) and approved by the Temple University Institutional Review Board (#24306). This study was carried out in accordance with the Declaration of Helsinki. Written informed consent was obtained from all subjects. The study was registered on ClinicalTrials.gov (NCT03243383), where the protocol is accessible.

### 2.2. Participants and Randomization

Inclusion criteria were an established diagnosis of diabetes, defined by preadmission use of a diabetes-specific medication and/or documentation of the diagnosis in the medical record, age ≥18 years, high predicted risk of 30-day readmission (≥27%) based on the DERRI^TM^ [6], and hospital admission to a non-critical care unit. Exclusion criteria were pregnancy, binge drinking (at least 5 alcoholic drinks for males or 4 alcoholic drinks for females on the same day), drug abuse within 3 months before admission, receiving palliative care during the hospitalization, participation in another readmission risk reduction program, planned or actual transfer to another hospital or subacute facility, discharge expected within 12 h, lack of access to a phone, living more than 30 miles away from the hospital, HbA1c <5.7% (39 mmol/mol), and inability to speak English. After enrollment, subjects were excluded upon transfer to another hospital or subacute facility, discharge to hospice or a long-term care facility, signing out against medical advice, or inpatient death. We screened a computer-generated list of patients who were admitted to non-critical care units with orders for routine point-of-care blood glucose testing. If the primary hospital team approved, then potentially eligible patients were approached in their hospital room for further screening and informed consent.

Subjects were randomly assigned with a computer-generated randomization scheme 1:1 in randomly permuted blocks of 2, 4, or 6 to receive either the Intervention or UC. The study statistician (H.Z.) generated the random allocation sequence. Group assignments were placed in sealed envelopes and revealed sequentially as subjects were randomized. Study coordinators enrolled participants and assigned them to interventions based on the allocation sequence.

### 2.3. Usual Care and Intervention

Subjects in the UC group received the standard discharge instructions, education, medication reconciliation, and follow-up according to routine practice. Discharge instructions were generated using the Epic Hyperspace^®^ (Verona, WI, USA) electronic health record (EHR), which is integrated between the inpatient and outpatient settings. Education was provided by bedside nurses and hospital providers at their discretion using stock materials in the EHR (ExitCare Clinical References). Subjects received training by a bedside nurse on using a glucometer and insulin as needed. Diabetes therapy upon discharge was determined by the primary team. Discharge instructions were routinely sent to the primary care provider (PCP) either by fax or EHR. Subjects received a phone call within 4 days after discharge from a hospital-employed community health worker that included checking on the health of the subject, confirming follow-up appointments and access to medications, and answering questions. Problems were referred to a nurse navigator, all of whom had a nurse practitioner degree, for further management. A transition-of-care appointment was scheduled for all patients with a PCP within 10 days of discharge. This appointment focused on medication reconciliation, review of discharge instructions, and updating care needs since hospital discharge.

Subjects in the Intervention group received the DiaTOHC Program in addition to the UC described above. The DiaTOHC Program has three components: (1) patient-centered discharge education, (2) HbA1c-based adjustment of diabetes therapy upon discharge, and (3) post-discharge support.

#### 2.3.1. Patient-Centered Discharge Education

The education consisted of two parts delivered by one of three study team navigators over the phone before discharge or 1 to 3 days after discharge according to subject availability. The first part was focused, customizable, diabetes discharge instructions and education using a 19-page booklet based on American Diabetes Association (ADA) guidelines that includes information on diet, physical activity, and self-care guidance, such as how to recognize and treat hypoglycemia and hyperglycemia [8]. The instructions addressing post-discharge use of diabetes medications were adapted from previously published work [9]. All the concepts tested in the revised Diabetes Knowledge Test (DKT2) are covered in the booklet [10]. Subjects who had not completed a formal outpatient diabetes education program in the prior 12 months were referred to a Diabetes Care and Education Specialist at the Temple Diabetes Center. The second part of discharge education was a comprehensive review of the discharge plan. A navigator reviewed the discharge plan with subjects, covering the treatment plan, how to take medications, reasons for and importance of follow-up appointments and testing, and how to reach post-hospital providers.

#### 2.3.2. HbA1c-Based Adjustment of Diabetes Therapy upon Discharge

Diabetes therapy upon hospital discharge was determined by a study endocrinologist (C.V. or D.R.) using an algorithm based on previously published work and ADA guidelines (Supplementary Table S1) [8,11]. For subjects with baseline HbA1c <7.0% (53 mmol/mol), the preadmission treatment regimen was continued unless adjustments were needed for safety. For subjects with baseline HbA1c >7.0% (53 mmol/mol), preadmission non-insulin diabetes therapy was optimized, defined as using the next higher dose up to the maximum tolerated dose. Only FDA-approved diabetes therapies were used in the study. Metformin was started in subjects with Type 2 Diabetes who did not have a contraindication to using it. Depending on the baseline HbA1c level, insulin was adjusted or added to the preadmission regimen.

#### 2.3.3. Post-Discharge Support

One to three days after discharge, a navigator called subjects to assess their status, confirm receipt of and compliance with medications, verify follow-up appointments, assess barriers to following the discharge plan, determine the need for a community health worker, and review BG levels. Similar phone calls were made weekly for four weeks following discharge or until the first unplanned readmission. Subjects who were discharged on non-insulin regimens were asked to check their BG levels at least once a day, and subjects discharged on insulin at least twice a day. If a subject reported BG levels <70 or >240 mg/dL (3.9 or 13.3 mmol/L), then the navigator notified a study physician, who contacted the subject by phone to adjust diabetes therapy per protocol (Supplementary Tables S2 and S3). In addition, all intervention subjects received a referral for a nursing visit in the home to assess medical needs for support at home. Referral to a community health worker was made if subjects were found to have non-medical needs and/or obstacles to maintaining self-care and attending follow-up appointments, including transportation, food, housing, financial, and legal issues.

### 2.4. Data Collection, Measures, and Sample Size

The primary outcome was all-cause unplanned hospital readmission within 30 days of discharge. Secondary outcomes assessed at 30 days after discharge were rate of any emergency department (ED) visit not associated with a hospital admission, composite rate of unplanned readmission or ED visit, cost of post-discharge acute care and the intervention (details below), daily frequency of self-monitored blood glucose (SMBG) testing, and three categories of hypoglycemia defined as any SMBG level <70, <54, or <40 mg/dL (3.9, 3, or 2.2 mmol/L, Intervention group only). Data on hypoglycemia in the Intervention group were obtained during the follow-up navigator phone calls. Because the UC group did not receive navigator calls, comparable data on hypoglycemia were not available. Baseline characteristics were recorded based on self-report and review of the medical record.

Approximately 5 weeks after discharge, a study coordinator called subjects in both groups to assess readmissions, ED visits, and the frequency of SMBG testing. In addition, a novel patient experience questionnaire was administered to Intervention subjects. Subjects were asked to respond to each of the following statements with either Agree, Neutral, or Disagree: (1) “I understood my discharge instructions,” (2) “The diabetes teaching in the booklet was helpful,” and (3) “I was happy with the support I got after leaving the hospital.” Healthcare encounters were confirmed in the EHR, which is integrated with several other healthcare systems in the region by Epic Care Everywhere. Readmissions that could not be confirmed in the EHR were confirmed by obtaining discharge records. Change in HbA1c level from baseline (hospital admission) was assessed 3 months after discharge.

The cost of post-discharge acute care was based on the sum of the estimated cost of all planned and unplanned readmissions and ED visits within 30 days of discharge. The cost of each readmission was based on the observed length of stay and a unit cost of USD 3045 per hospital day in 2017 among patients with diabetes [12]. The cost of each ED visit was based on a unit cost of USD 1110 [12]. The cost of the intervention was based on the value of time spent by the navigators and study physicians. Based on the average annual income of a navigator working 2000 h per year, navigator time was valued at USD 58 per hour. Similarly, based on the fiscal year 2018 median income of an assistant professor in endocrinology working 2300 h per year in the United States, physician time was valued at USD 101 per hour [13].

A target of 60 subjects per group was the largest deemed feasible. The last date of enrollment was determined by navigator availability. As a pilot trial, this study is not powered to detect statistically significant differences in outcomes.

### 2.5. Analysis

Distributions of the data were assessed by descriptive statistical and graphical methods. Summary statistics are reported as mean ± standard deviation or median (interquartile range). Because of skewed distributions, the ratio of estimated costs between groups was calculated using log-transformed gamma regression [14]. The primary analyses for all outcomes were performed in the intention-to-treat (ITT) population, defined as having been randomly assigned to a study group and not meeting post-enrollment exclusion criteria. In prespecified analyses, outcomes were assessed in the ITT subgroup of subjects with a baseline HbA1c >7.0% (53 mmol/mol). No statistical testing was performed for this pilot trial.

## 3. Results

### 3.1. Participant Flow

**Figure 1:**
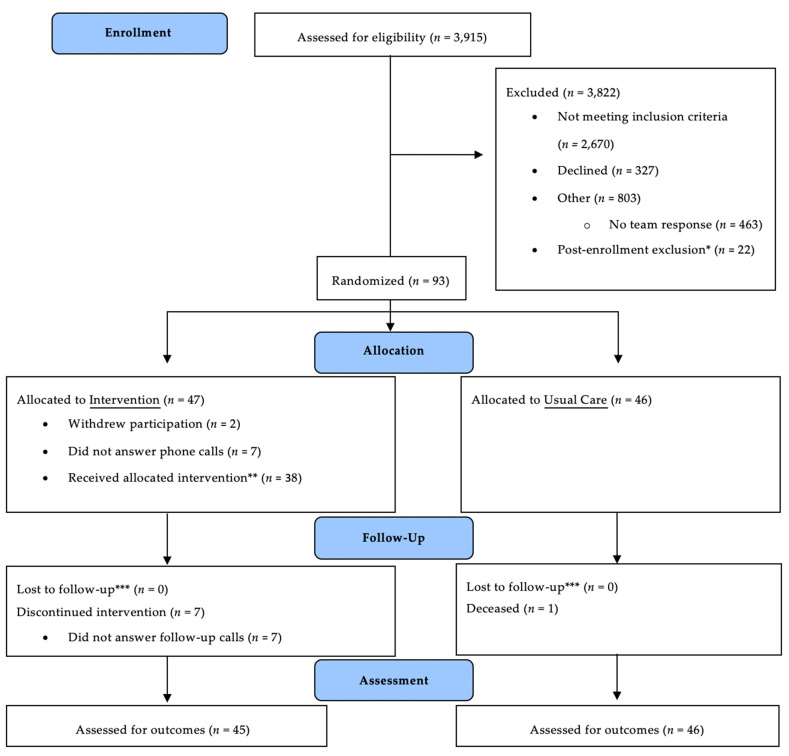
Flow of patients in trial. * Post-enrollment exclusion criteria were transfer to another hospital or subacute facility, discharge to hospice or a long-term care facility, signing out against medical advice, or inpatient death; ** Subject received education, adjustment of diabetes therapy upon discharge, and at least 1 follow-up phone call; *** Electronic health record used for follow-up if subject could not be contacted.

Between 16 October 2017 and 30 May 2019, a total of 3915 patients were assessed for eligibility and 3822 were excluded (Figure 1). The remaining 93 patients were randomized, and 47 were allocated to Intervention, 46 to UC. Because two subjects withdrew consent, the analyzed ITT cohort had 45 Intervention subjects and 46 UC subjects.

### 3.2. Baseline Characteristics

Mean age was 58.7 ± 12.7 years, duration of diabetes 15.1 ± 10.0 years, and median HbA1c 8.7% (7.1–10.6%), 72 mmol/mol (54–92 mmol/mol) (Table 1). The cohort was 71% Black, 28% White, 14% Hispanic and mostly low-income (86%). Most patients (95%) had Type 2 Diabetes. Predicted 30-day readmission risk was similar between groups (38.4 ± 7.6% Intervention, 37.5 ± 7.5% UC).

### 3.3. Outcomes

The 30-day readmission rate was 31.1% in the Intervention group and 32.6% in the UC group (Table 2). The combined 30-day readmission or ED visit rate was 35.6% in the Intervention group and 39.1% in the UC group. The number of SMBG tests was 2.4 ± 1.6 per day in the Intervention group and 1.8 ± 1.4 per day in the UC group. Costs in the Intervention group were 33% of the costs in the UC group. Only 11% of Intervention participants reported having at least one BG level <70 mg/dL (3.9 mmol/L) during follow-up. Change in HbA1c was similar at 3 months between the two groups. Among survey respondents in the Intervention group, 97% understood their discharge instructions, 93% believed the diabetes teaching was helpful, and 93% were happy with the support they received after leaving the hospital.

### 3.4. Ancillary Analysis

Among the 69 subjects with baseline HbA1c >7.0% (53 mmol/mol), the 30-day readmission rate was 23.5% in the Intervention group and 31.4% in the UC group (Table 2). The combined 30-day readmission or ED visit rate was 26.5% in the Intervention group and 40.0% in the UC group. Among the Intervention participants, 15% reported having at least one BG level <70 mg/dL (3.9 mmol/L) during follow-up. The number of SMBG tests was 2.5 ± 1.6 per day in the Intervention group and 2.0 ± 1.5 per day in the UC group. Costs in the Intervention group were 21% of the costs in the UC group. Change in HbA1c was similar at 3 months between the two groups.

## 4. Discussion

This pilot RCT suggests the DiaTOHC intervention, with which participants were overwhelmingly satisfied, may be feasible at an urban, academic, safety-net hospital. Readmission rates in the Intervention and UC groups were similar. However, the trial raises the possibility that the intervention may decrease readmission/ED visit risk among patients with a baseline HbA1c >7.0% (53 mmol/mol). In this subgroup, Intervention subjects experienced a 34% relative risk reduction in readmission/ED visit risk and absolute risk reduction of 13.5%. Additionally, costs were substantially lower in the Intervention group. Furthermore, hypoglycemia during the intervention was uncommon, with 11% of Intervention participants reporting any SMBG <70 mg/dL (3.9 mmol/L). Other trials with similar HbA1c-based discharge treatment algorithms reported post-discharge hypoglycemia rates of 23–29% [11,15].

Several mostly observational studies have investigated the effect of various interventions on readmission risk in diabetes patients [4], categorizable as inpatient diabetes education only, inpatient diabetes management by a dedicated service, and multi-component programs consisting of education, transition-of-care support, and/or outpatient follow-up. The relative risk reductions of these interventions vary considerably from 0 to 71%, with most studies showing benefit.

The current study adds to the small number of related published RCTs with a novel approach: combining multi-component intervention with selection of high-risk patients using a validated tool. This pilot study, however, is limited by lacking power for detecting differences between groups. Because observation of the UC group was limited, we were unable to compare hypoglycemia rates or office visits between groups. Given the nature of the intervention, blinding was not feasible. In addition, the statisticians were not blinded. Lastly, the findings may not generalize to other sites and settings.

In conclusion, the possible reduction in 30-day readmission/ED visit risk in the higher HbA1c subgroup merits further investigation in a larger, multi-center RCT.

## Figures and Tables

**Table 1:** Baseline characteristics of Intervention and Usual Care groups.

Variable	All Patients *N* = 91	Intervention *n* = 45	Usual Care *n* = 46
Age, years	58.7 ± 12.7	58.5 ± 13.7	58.9 ± 11.7
Female	47 (51.6)	21 (46.7)	26 (56.5)
Income, USD			
Less than $12,060	25 (27.5)	9 (20.0)	16 (34.8)
$12,060–$16,239	16 (17.6)	8 (17.8)	8 (17.4)
$16,240–$24,599	15 (16.5)	9 (20.0)	6 (13.0)
$24,600–$49,999	22 (24.2)	11 (24.4)	11 (23.9)
$50,000 or more	13 (14.3)	8 (17.8)	5 (10.9)
Race			
Black	65 (71.4)	29 (64.4)	36 (78.3)
Other	1 (1.1)	0 (0.0)	1 (2.2)
White	25 (27.5)	16 (35.6)	9 (20.0)
Hispanic	13 (14.3)	9 (20.0)	4 (8.7)
Education, years	12.6 ± 2.5	13.0 ± 3.0	12.1 ± 1.8
Employment Status			
Disabled	64 (70.3)	32 (71.1)	32 (69.6)
Employed	1 (1.1)	1 (2.2)	0 (0.0)
Retired	16 (17.6)	6 (13.3)	10 (21.7)
Unemployed	10 (11.0)	6 (13.3)	4 (8.7)
Insurance			
Medicaid only	16 (18.0)	9 (20.9)	7 (15.2)
Medicare and Medicaid	17 (19.1)	9 (20.9)	8 (17.4)
Medicare only	24 (27.0)	10 (23.3)	14 (30.4)
None	3 (3.4)	2 (4.7)	1 (2.2)
Private	29 (32.6)	13 (30.2)	16 (34.8)
Smoking			
Current smoker	18 (19.8)	9 (20.0)	9 (19.6)
Former smoker	40 (44.0)	20 (44.4)	20 (43.5)
Never	33 (36.3)	16 (35.6)	17 (37.0)
Body mass index (kg/m^2^)	35.2 ± 10.9	36.2 ± 11.7	34.2 ± 10.0
Type of Diabetes			
Type 1	5 (5.5)	3 (6.7)	2 (4.3)
Type 2	86 (94.5)	42 (93.3)	44 (95.7)
Diabetes duration, years	15.1 ± 10.0	13.6 ± 8.5	16.6 ± 11.2
A1c at admission	8.7 (7.1–10.6)	8.9 (7.2–11.1)	8.5 (7.1–10.0)
A1c at admission >7.0% (53 mmol/mol)	69 (76.7)	34 (77.3)	35 (76.1)
Preadmission Home Medication Route			
Insulin only	52 (57.1)	27 (60.0)	25 (54.3)
No medications	7 (7.7)	1 (2.2)	6 (13.0)
Oral & insulin	19 (20.9)	13 (28.9)	6 (13.0)
Oral only	11 (12.1)	3 (6.7)	8 (17.4)
Other	2 (2.2)	1 (2.2)	1 (2.2)
Preadmission sulfonylurea use	8 (8.8)	3 (6.7)	5 (10.9)
Preadmission metformin use	19 (20.9)	9 (20.0)	10 (21.7)
Preadmission insulin use	73 (80.2)	42 (93.3)	31 (67.4)
Preadmission statin use	64 (70.3)	28 (62.2)	36 (78.3)
Preadmission glucocorticoid use	18 (19.8)	8 (17.8)	10 (21.7)
Preadmission blood pressure medications			
None	14 (15.4)	9 (20.0)	5 (10.9)
ACE-i or ARB and Non-ACE/ARB	25 (27.5)	13 (28.9)	12 (26.1)
Only ACE-i or ARB	23 (25.3)	6 (13.3)	17 (37.0)
Only non-ACE or ARB	29 (31.9)	17 (37.8)	12 (26.1)
History of severe hypoglycemia	34 (37.8)	17 (37.8)	17 (37.8)
Current or prior DKA or HHS	9 (9.9)	5 (11.1)	4 (8.7)
Microvascular complications			
0	35 (38.5)	15 (33.3)	20 (43.5)
1	35 (38.5)	20 (44.4)	15 (32.6)
2	15 (16.5)	7 (15.6)	8 (17.4)
3	6 (6.6)	3 (6.7)	3 (6.5)
Macrovascular complications			
0	25 (27.5)	13 (28.9)	12 (26.1)
1	38 (41.8)	20 (44.4)	18 (39.1)
2	21 (23.1)	9 (20.0)	12 (26.1)
3	6 (6.6)	2 (4.4)	4 (8.7)
4	1 (1.1)	1 (2.2)	0 (0.0)
Anemia diagnosis	62 (68.1)	33 (73.3)	29 (63.0)
Discharged within 90 days before index admission	81 (89.0)	45 (100.0)	36 (78.3)
ED visit within 90 days before index admission	24 (30.4)	10 (26.3)	14 (34.1)
Admission priority			
Emergent	75 (82.4)	37 (82.2)	38 (82.6)
Planned	4 (4.4)	2 (4.4)	2 (4.3)
Urgent	12 (13.2)	6 (13.3)	6 (13.0)
Home zip code within 5 miles of hospital	78 (85.7)	40 (88.9)	38 (82.6)
Discharge status			
Against medical advice	1 (1.1)	0 (0.0)	1 (2.2)
Home with nursing care	28 (30.8)	14 (31.1)	14 (30.4)
Home without additional services	56 (61.5)	29 (64.4)	27 (58.7)
Subacute facility (rehabilitation or skilled nursing)	5 (5.5)	2 (4.4)	3 (6.5)
No discharge within prior year	1 (1.1)	0 (0.0)	1 (2.2)
Predicted risk of readmission within 30 days, %	38.4 ± 7.6	39.2 ± 7.8	37.5 ± 7.5
Admission blood glucose, mg/dL	208.1 ± 107.7	188.7 ± 95.6	227.1 ± 116.4
Admission blood glucose, mmol/L	11.6 ± 6.0	10.5 ± 5.3	12.6 ± 6.5
Admission serum sodium, mmol/L	136.0 ± 4.9	136.3 ± 4.9	135.7 ± 5.0
Admission serum potassium, mmol/L	4.3 ± 0.8	4.3 ± 0.9	4.2 ± 0.7
Admission serum creatinine, mg/dL	1.7 (1.1–3.2)	2.0 (1.1–3.2)	1.5 (1.1–3.2)
Admission eGFR, mL/min	39.8 ± 20.6	39.8 ± 20.5	39.8 ± 20.9
Admission hematocrit, %			
High	2 (2.2)	2 (4.4)	0 (0.0)
Low	69 (75.8)	30 (66.7)	39 (84.8)
Normal	20 (22.0)	13 (28.9)	7 (15.2)
Brief Health Literacy Screen Score	11.9 ± 2.9	12.3 ± 3.0	11.6 ± 2.7
PHQ–2 Score	1.0 (0.0–2.0)	1.0 (0.0–2.0)	2.0 (1.0–3.0)
Diabetes Knowledge Test Score	57.3 ± 15.6	59.1 ± 15.7	55.5 ± 15.5
Problem Areas in Diabetes Score	30.6 ± 24.3	36.3 ± 25.1	25.1 ± 22.4
Predicted risk of readmission within 30 days, % *	38.4 ±7.6	39.2 ± 7.8	37.5 ± 7.5

Values are mean ± SD, median (IQR), or *n* (%) unless otherwise stated. * Predicted risk based on Diabetes Early Readmission Risk Indicator (DERRI^TM^). IQR (interquartile range), ACE-i (angiotensin-converting enzyme inhibitors), ARB (angiotensin II receptor blockers), DKA (diabetic ketoacidosis), HHS (hyperosmolar hyperglycemic state), eGFR (estimated glomerular filtration rate), PHQ (patient health questionnaire).

**Table 2:** Outcomes in Intervention and Usual care groups.

Intention-to-Treat Cohort			
Variable ^a^	All Patients *N* = 91	Intervention *n* = 45	Usual Care *n* = 46
Readmission	29 (31.9)	14 (31.1)	15 (32.6)
ED visit	8 (8.8)	4 (8.9)	4 (8.7)
Readmission or ED visit	34 (37.4)	16 (35.6)	18 (39.1)
Costs, USD	--	5542 ± 10,970	6657 ± 16,969
Costs, USD	--	172 (127–5546)	0 (0–5667)
Costs, Intervention:Usual Care ratio ^b^ (95%CI)	0.33 (0.13–0.79)		
Hypoglycemia			
-Blood glucose <70 mg/dL (3.9 mmol/L)	--	5 (11)	--
-Blood glucose <54 mg/dL (3.0 mmol/L)	--	2 (4)	--
-Blood glucose <40 mg/dL (2.2 mmol/L)	--	1 (2)	--
Number of daily SMBG tests	2.1 ± 1.5	2.4 ± 1.6	1.8 ± 1.4
Change in HbA1c at 3 months, %	−0.9 (−1.6–0.2)	−1.0 (−1.6–0.2)	−0.9 (−1.4–0.2)
Change in HbA1c at 3 months, mmol/mol	−10 (−18–2)	−11 (−18–2)	−10 (−15–2)
Subgroup with baseline HbA1c >7.0%	All Patients *N* = 69	Intervention *n* = 34	Usual Care *n* = 35
Readmission	19 (27.5)	8 (23.5)	11 (31.4)
ED visit	7 (10.1)	3 (8.8)	4 (11.4)
Readmission or ED visit	23 (33.3)	9 (26.5)	14 (40.0)
Costs, USD	--	3657 ± 8230	6967 ± 18,863
Costs, USD	--	154 (126–1246)	0 (0–5661)
Costs, Intervention:Usual Care ratio ^b^ (95%CI)	0.21 (0.08–0.58)		
Hypoglycemia			
-Blood glucose <70 mg/dL (3.9 mmol/L)	--	5 (14.7)	--
-Blood glucose <54 mg/dL (3.0 mmol/L)	--	2 (5.9)	--
-Blood glucose <40 mg/dL (2.2 mmol/L)	--	1 (2.9)	--
Number of daily SMBG tests	2.2 ± 1.6	2.5 ± 1.6	2.0 ± 1.5
Change in HbA1c at 3 months, %	−1.0 (−2.2–0.0)	−1.1 (−2.2–0.0)	−0.9 (−2.3–0.1)
Change in HbA1c at 3 months, mmol/mol	−11 (−24–0)	−12 (−24–0)	−10 (−25–1)

Values are mean ± SD, median (IQR), or *n* (%) unless otherwise stated. IQR, interquartile range; CI, confidence interval. ^a^ Within the 30 days after hospital discharge. ^b^ Costs of 30-day readmissions, ED visits, and the intervention. SMBG, self-monitored blood glucose.

## Data Availability

Data are available on request due to restrictions on sharing of protected health information.

## Supplementary Materials

The following supporting information can be downloaded at: https://www.mdpi.com/article/10.3390/jcm11061471/s1, Table S1: HbA1c-based adjustment of diabetes therapy; Table S2: Outpatient basal insulin dose adjustment; Table S3: Outpatient prandial/pre-meal insulin dose adjustment based on subsequent mealtime/HS BG values [16].
