# Supplementary material for: The Diabetes Transition of Hospital Care (DiaTOHC) Pilot Study: A Randomized Controlled Trial of an Intervention Designed to Reduce Readmission Risk of Adults with Diabetes
Source: J Clin Med. 2022 Mar 8;11(6):1471. doi: 10.3390/jcm11061471 (PMC8949063; doi:10.3390/jcm11061471)
Supplement: Supplementary file 1 [file jcm-11-01471-s001.zip › jcm-1564895-supplementary.pdf]

Supplemental Table S1. HbA1c-based adjustment of diabetes therapy

a) For HbA1c <7.0% (53 mmol/mol), resume the pre-admission treatment regimen.

b) For HbA1c 7.0 to 7.9% (53-63 mmol/mol)

- Patients who did not take insulin before admission, discharge on optimized pre-admission treatment regimen (see definition below) or add a non-insulin agent if the prior regimen was already optimal.
- Patients who took basal insulin but not prandial insulin before admission, increase the home daily dose of basal insulin by 10-15% in addition to any non-insulin pre-admission treatments.
- Patients who took multiple daily insulin injections (MDI) before admission, increase the home total daily dose of insulin by 10-15% in addition to any non-insulin pre-admission treatments.

c) For HbA1c 8.0 to 9.0% (64-75 mmol/mol)

- Patients who did not take insulin before admission, discharge on 50% of the last inpatient insulin glargine daily dose or 0.2 units/kg in addition to the pre-admission treatment regimen, which should be optimized.
- Patients who took basal insulin but not prandial insulin before admission, discharge on 50-80% of the last inpatient insulin glargine daily dose or increase the home daily dose of basal insulin by 10-15% and/or add rapid-acting insulin before the largest meal at 50-80% of the last inpatient dose or 0.1 units/kg in addition to any non-insulin pre-admission treatments.
- Patients who took multiple daily insulin injections (MDI) before admission, discharge on 50-80% of the last inpatient total daily insulin dose or increase the home total daily dose of insulin by 10-15% in addition to any non-insulin pre-admission treatments.

d) For HbA1c >9.0% (75 mmol/mol)

- Patients who did not take insulin before admission, discharge on 80-100% of the last inpatient insulin glargine daily dose or 0.3 units/kg in addition to the pre-admission treatment regimen, which should be optimized.
- Patients who took basal insulin but not prandial insulin before admission, discharge on 80-100% of the last inpatient insulin glargine daily dose or increase the home daily dose of basal insulin by 20-30% and/or add rapid-acting insulin before the largest meal at 80-100% of the last inpatient dose or 0.1 units/kg in addition to any non-insulin pre-admission treatments.
- Patients who took multiple daily insulin injections (MDI) before admission, discharge on 80-100% of the last inpatient total daily insulin dose or increase the home total

daily dose of insulin by 20-30% in addition to any non-insulin pre-admission treatments.

For all subjects with baseline HbA1c >7.0% (53 mmol/mol), non-insulin diabetes therapy was optimized, defined as using the next higher dose up to the maximum tolerated dose. Only FDA-approved diabetes therapies were used in the study.

Supplemental Table S2. Outpatient basal insulin dose adjustment

**Fasting blood glucose**	**Basal insulin dose adjustment**
If mean **FBG > 180 mg/dL** for the last 2 consecutive days and no episodes of hypoglycemia	Increase daily basal dose by 4 U
If mean **FBG > 140 mg/dL** for the last 2 consecutive days and no episodes of hypoglycemia	Increase daily basal dose by 2 U
If mean **FBG between 100 to 140 mg/dL** for the last 2 consecutive days and no episodes of hypoglycemia	No Change
If any FBG between 70 – 99 mg/dl	Decrease by 4 U or 10% of total daily basal dose
If any FBG < 70 mg/dl	Decrease by 8 U or 20% of total daily basal dose
If any FBG < 40 mg/dl	Decrease total daily basal dose by 30%

FBG=Fasting blood glucose; Hypoglycemia=typical symptoms (e.g., sweating, tremor, acute hunger, anxiety) and/or blood glucose <70 mg/dL

Supplemental Table S3. Outpatient prandial/pre-meal insulin dose adjustment based on subsequent mealtime/HS BG values

**Pre-meal Dose, U**	**BG 70 – 100 mg/dl***	**BG 141-180 mg/dl****
≤ 10 U	Decrease by 1 U	Increase by 1 U
>11- 19 U	Decrease by 2 U	Increase by 2 U
≥ 20 U	Decrease by 3 U	Increase by 3 U
**Pre-meal Dose, U**	**BG 40-70 mg/dl x 1**	**BG 180-240 mg/dl x 1**
≤ 10 U	Decrease by 2 U	Increase by 2 U
>11- 19 U	Decrease by 3 U	Increase by 3 U
≥ 20 U	Decrease by 4 U	Increase by 4 U
**Pre-meal Dose, U**	**BG < 40 mg/dl x 1*****	**BG > 240 mg/dl x 1**
≤ 10 U	Decrease by 4 U	Increase by 3 U
>11- 19 U	Decrease by 6 U	Increase by 4 U
≥ 20 U	Decrease by 8 U	Increase by 5 U

Pre-meal insulin dose adjustment is based on the subsequent BG value, e.g., pre-breakfast insulin dose is based on the pre-lunch BG.

* If > ½ of the mealtime/HS BG values for the week were below target.
**If > ½ of the mealtime/HS BG values for the week were above target.
*** Decrease by 30-40% in the event of severe hypoglycemia (mealtime/HS BG < 40 mg/dl).
BG=blood glucose; Mealtime/HS=pre-lunch, pre-dinner, or bedtime

The above algorithm provides recommended insulin doses and may have been modified based on clinical judgment of the investigator or co-investigator.

CONSORT 2010 checklist of information to include when reporting a pilot or feasibility trial

Section/Topic	Item No	Checklist item	Reported on page No
**Title and abstract**			
	1a	Identification as a pilot or feasibility randomised trial in the title	1
	1b	Structured summary of pilot trial design, methods, results, and conclusions (for specific guidance see CONSORT abstract extension for pilot trials)	1
**Introduction**			
Background and objectives	2a	Scientific background and explanation of rationale for future definitive trial, and reasons for randomised pilot trial	2
	2b	Specific objectives or research questions for pilot trial	2
**Methods**			
Trial design	3a	Description of pilot trial design (such as parallel, factorial) including allocation ratio	2
	3b	Important changes to methods after pilot trial commencement (such as eligibility criteria), with reasons	n/a
Participants	4a	Eligibility criteria for participants	2
	4b	Settings and locations where the data were collected	2
	4c	How participants were identified and consented	2
Interventions	5	The interventions for each group with sufficient details to allow replication, including how and when they were actually administered	2-4
Outcomes	6a	Completely defined prespecified assessments or measurements to address each pilot trial objective specified in 2b, including how and when they were assessed	4
	6b	Any changes to pilot trial assessments or measurements after the pilot trial commenced, with reasons	None

	6c	If applicable, prespecified criteria used to judge whether, or how, to proceed with future definitive trial	n/a
Sample size	7a	Rationale for numbers in the pilot trial	4
	7b	When applicable, explanation of any interim analyses and stopping guidelines	n/a
Randomisation:			
Sequence generation	8a	Method used to generate the random allocation sequence	2
	8b	Type of randomisation(s); details of any restriction (such as blocking and block size)	2
Allocation concealment mechanism	9	Mechanism used to implement the random allocation sequence (such as sequentially numbered containers), describing any steps taken to conceal the sequence until interventions were assigned	2
Implementation	10	Who generated the random allocation sequence, who enrolled participants, and who assigned participants to interventions	2
Blinding	11a	If done, who was blinded after assignment to interventions (for example, participants, care providers, those assessing outcomes) and how	n/a
	11b	If relevant, description of the similarity of interventions	n/a
Statistical methods	12	Methods used to address each pilot trial objective whether qualitative or quantitative	4-5
**Results**			
Participant flow (a diagram is strongly recommended)	13a	For each group, the numbers of participants who were approached and/or assessed for eligibility, randomly assigned, received intended treatment, and were assessed for each objective	5
	13b	For each group, losses and exclusions after randomisation, together with reasons	5
Recruitment	14a	Dates defining the periods of recruitment and follow-up	5
	14b	Why the pilot trial ended or was stopped	4
Baseline data	15	A table showing baseline demographic and clinical characteristics for each group	6-8

Numbers analysed	16	For each objective, number of participants (denominator) included in each analysis. If relevant, these numbers should be by randomised group	9
Outcomes and estimation	17	For each objective, results including expressions of uncertainty (such as 95% confidence interval) for any estimates. If relevant, these results should be by randomised group	9
Ancillary analyses	18	Results of any other analyses performed that could be used to inform the future definitive trial	9-10
Harms	19	All important harms or unintended effects in each group (for specific guidance see CONSORT for harms)	9
	19a	If relevant, other important unintended consequences	n/a
**Discussion**			
Limitations	20	Pilot trial limitations, addressing sources of potential bias and remaining uncertainty about feasibility	10-11
Generalisability	21	Generalisability (applicability) of pilot trial methods and findings to future definitive trial and other studies	12
Interpretation	22	Interpretation consistent with pilot trial objectives and findings, balancing potential benefits and harms, and considering other relevant evidence	10-11
	22a	Implications for progression from pilot to future definitive trial, including any proposed amendments	11
**Other information**			
Registration	23	Registration number for pilot trial and name of trial registry	2
Protocol	24	Where the pilot trial protocol can be accessed, if available	2
Funding	25	Sources of funding and other support (such as supply of drugs), role of funders	11
	26	Ethical approval or approval by research review committee, confirmed with reference number	2 and 11

Citation: Eldridge SM, Chan CL, Campbell MJ, Bond CM, Hopewell S, Thabane L, et al. CONSORT 2010 statement: extension to randomised pilot and feasibility trials. BMJ. 2016;355.
